# Blast Clearance Dynamics and Time to Response Across *IDH1‐* and *IDH2*‐Mutated AML

**DOI:** 10.1002/jha2.70204

**Published:** 2025-12-15

**Authors:** Mohamad Sheikh Najeeb, Oudai Alkabbani, Rong He, Dragan Jevremovic, Patricia T. Greipp, Aasiya Matin, Mehrdad Hefazi, Abishek Mangaonkar, Mrinal Patnaik, Naseema Gangat, William J. Hogan, Mark R. Litzow, Cecila Y. Arana Yi, James M. Foran, Hassan B Alkhateeb, Mithun Shah, Kebede Begna, Tariq Kewan, Antoine N. Saliba, Aref Al‐Kali

**Affiliations:** ^1^ Division of Hematology Mayo Clinic Rochester Minnesota USA; ^2^ Division of Hematopathology Mayo Clinic Rochester Minnesota USA; ^3^ Division of Hematology Mayo Clinic Phoenix Arizona USA; ^4^ Division of Hematology Mayo Clinic Jacksonville Florida USA

**Keywords:** AML, *IDH1*, *IDH2*, prognosis, response

## Abstract

**Background:**

*IDH* mutations are found in 15%–20% of acute myeloid leukemia (AML). Recent evidence suggests that *IDH* mutation subtypes may respond differently to intensive chemotherapy. We evaluated blast clearance patterns, remission outcomes, and overall survival (OS) in AML patients with *IDH2* R140, *IDH2* R172, and *IDH1* R132 mutations to assess differences in treatment response.

**Methods:**

We retrospectively reviewed AML patients diagnosed at Mayo Clinic, Rochester (2016–2023) with NGS data and treated with intensive chemotherapy (7+3 or CPX‐351). Bone marrow blasts were assessed at diagnosis, mid‐induction, and end‐of‐cycle; blast clearance was defined as < 5%. Composite remission (CRc) included CR/CRi per ELN 2022. *IDH2* responders were classified as early (after Cycle 1) or late (≥ 2 cycles). Blast reduction was calculated as log_10_ (diagnosis blasts/end‐induction blasts) and normalized per day. OS was measured from diagnosis.

**Results:**

Among 381 patients, 31 had *IDH2* mutations (23 R140, 8 R172) and eight had *IDH1* R132. Baseline blasts were similar. Mid‐cycle blasts were lower in R140 versus R172 (4% vs. 10%), with higher clearance (68% vs. 38%). End‐induction blasts were lower in R140 versus R172 (2% vs. 4%) with higher clearance (100% vs. 75%). *IDH1* and *IDH2* showed similar clearance patterns. Log_10_ analyses showed deeper and faster blast reduction in R140 versus R172, with comparable kinetics between *IDH1* and *IDH2*. OS did not differ across subtypes. Early CRc in *IDH2*‐mutated patients was associated with numerically longer OS.

**Conclusion:**

*IDH2* R140 demonstrated faster blast clearance than R172, while remission and survival were similar across *IDH* subtypes.

**Trial Registration**: The authors have confirmed clinical trial registration is not needed for this submission.

1

To the Editor;

Acute myeloid leukemia (AML) is characterized by a rapid clonal expansion of undifferentiated myeloid precursors, disrupting normal hematopoiesis and resulting in bone marrow failure [1]. The prognosis of AML is variable and shaped by patient characteristics such as age, performance status, and comorbidities, as well as leukemia‐specific genetic features, including cytogenetics and molecular classification [2]. Recurring mutations in *isocitrate dehydrogenase* (*IDH*) genes are detected in approximately 20% of adult patients with AML, commonly at codon R132 in *IDH1* and R140 or R172 in *IDH2* [3, 4]. Recent evidence suggests that *IDH2* variants may exhibit differential response kinetics to intensive chemotherapy, with R172‐mutated AML showing slower blast clearance than R140‐mutated disease [5]. In this study, we compared blast clearance dynamics and remission outcomes among intensively treated patients with AML with *IDH2* R140 and *IDH2* R172 mutations, and further between *IDH1* and *IDH2* mutations in a single‐center AML cohort.

We reviewed the charts of AML patients diagnosed at Mayo Clinic, Rochester, between the years 2016 and 2023 that had available next‐generation sequencing (NGS) data. Institutional review board approval was obtained. All data were recorded at the time of AML diagnosis. Patients without molecular data or not treated with intensive chemotherapy (7+3 or CPX‐351‐based regimens) were excluded from this study. Bone marrow blast percentages were assessed at diagnosis, mid‐induction (Days 14–21), and end of induction (count recovery). Blast clearance was defined as < 5% bone marrow blasts. Composite remission (CRc) rate included complete remission (CR) and CR with incomplete hematologic recovery (CRi) per the 2022 European LeukemiaNet (ELN) response criteria [6]. *IDH2*‐mutated patients achieving CRc were classified as early if remission occurred after the first induction cycle and late if after ≥ 2 cycles. Overall survival (OS) was measured from diagnosis to death, with censoring at last follow‐up. To quantify the magnitude and kinetics of blast reduction, we calculated the log_10_ fold reduction in blasts as log_10_ (blast [%] at diagnosis/blast [%] at end‐induction) for each patient, comparing *IDH* subgroups. Patients who changed regimens or discontinued induction early were analyzed using their last available marrow prior to therapy change. In addition, the rate of blast clearance was calculated as the log_10_ fold reduction divided by the number of days from diagnosis to response assessment (log_10_ per day), using an intention‐to‐treat approach in which non‐responders were assigned a log_10_ fold reduction of zero. GraphPad Prism 10.0.0 was used for statistical analysis.

Out of 381 AML patients who met our criteria, 31 had *IDH2* mutations, including 23 with R140 (74%) and 8 with R172 (26%), and 8 had *IDH1* mutations (all R132). The most common mutations in *IDH2* patients were *NPM1* (32%), *DNMT3A* (32%), and *RUNX1* (26%) (Table S1). At diagnosis, bone marrow blast percentages did not differ significantly between *IDH2‐R140* and *IDH2‐R172* subgroups (median 68% vs. 71%, *p* = 0.83, Figure S1). Similarly, no difference was observed when comparing patients with *IDH1* versus *IDH2* mutations (median 71% vs. 69%, *p* = 0.88, Figure S2). Among patients with *IDH2* mutations, those with R172 had numerically higher median mid‐cycle bone marrow blast percentage compared to R140 (10% vs. 4%, *p* = 0.36, Figure S3). There was no significant difference in mid‐cycle blast percentages between patients with *IDH1* and *IDH2* mutations (4.5% vs. 4%, *p* = 0.8, Figure S4). At mid‐cycle, 68% of patients with *IDH2* R140 had blast counts < 5%, compared to 38% of those with *IDH2* R172, suggesting a trend toward faster mid‐cycle clearance in the R140 group, though not statistically significant (*p* = 0.2, Figure S5). In a broader comparison, 60% of *IDH2*‐mutated patients and 50% of *IDH1*‐mutated patients achieved blast clearance (blasts < 5%) at mid‐cycle, though not statistically significant (*p* = 0.69, Figure S6).

At the end of induction, patients with *IDH2* R140 had lower median bone marrow blast percentages compared to those with R172 mutations (2% vs. 4%, *p* = 0.01, Figure S7). Similarly, patients with *IDH1* mutations showed low blast counts compared to the *IDH2* group (1.5% vs. 3%, *p* = 0.1, Figure S8). A higher proportion of *IDH2* R140 patients achieved blast clearance compared to those with R172 mutations (100% vs. 75%, *p* = 0.06, Figure S9), while blast clearance was similar between *IDH1* and *IDH2* patients (87.5% vs. 93.1%, *p* = 0.5, Figure S10).

To further characterize response kinetics, we compared the magnitude and rate of blast reduction across *IDH* mutation subgroups on a log_10_ scale. Among *IDH2*‐mutated patients, the median log_10_ fold reduction in blasts from diagnosis to end of induction was greater in *IDH2*‐R140 compared to *IDH2*‐R172 (1.37 vs. 1.21, *p* = 0.28), indicating a trend toward deeper blast clearance in the R140 subgroup (Figure S11). Similarly, the rate of blast reduction, calculated as log_10_ fold reduction per day, was numerically faster in *IDH2*‐R140 than *IDH2*‐R172 (4% vs. 2.5% log_10_/day, *p* = 0.30), suggesting a tendency toward more rapid blast cytoreduction in R140‐mutated AML (Figure S12).

When comparing *IDH1* and *IDH2* subtypes, median log_10_ fold reduction was 1.63 for *IDH1* and 1.35 for *IDH2* (*p* = 0.2317), while the median rate of blast reduction was 4.5% versus 4% log_10_/day (*p* = 0.1429), respectively, indicating comparable magnitude and kinetics of blast clearance between *IDH1*‐ and *IDH2*‐mutated AML (Figures S13 and S14).

Within the *IDH2*‐mutated group, the R140 subgroup had a numerically higher CRc rate compared to the R172 subgroup (87% vs. 75, *p* = 0.5, Figure S15). Similarly, when comparing *IDH1*‐ and *IDH2*‐mutated patients, CRc rates were 75% and 84%, respectively, with no significant difference observed (*p* = 0.6, Figure S16). Kaplan–Meier analysis of time to end of induction blast clearance revealed no statistically significant difference between *IDH2* R140 and R172 patients (median time to clearance: 31 vs. 37 days, *p* = 0.3, Figure S17). Similarly, no difference was observed between *IDH1* and *IDH2*‐mutated patients (33 vs. 31 days, *p* = 0.9, Figure S18).

To evaluate whether the timing of CR influenced outcomes, *IDH2*‐mutated patients achieving CRc were categorized as early or late responders. The mean OS was 61.8 months in the early CRc group and 33.8 months in the late CRc group. The median OS was not reached in the early CRc group and was 16.6 months in the late CRc group (*p* = 0.4, log‐rank test), suggesting a trend toward improved survival among early responders (Figure S19).

OS analyses showed no significant difference between *IDH2* R140 and R172 groups (median 66.8 months vs. not reached, *p* = 0.4, Figure S20). Likewise, no difference was observed between *IDH1* and *IDH2* patients (44.4 months vs. not reached, *p* = 0.7), indicating similar OS across mutation subtypes (Figure S21).

Analysis of ELN 2022 risk classification distribution showed no significant difference between *IDH2*‐R140 (22% favorable, 26% intermediate, and 52% adverse) and *IDH2*‐R172 (13%, 38%, 50%, respectively) (*p* = 0.77; Figure S22). Results of the study analyses are summarized in Table 1.

**TABLE 1:** Response outcomes by *IDH* mutation subtype in AML.

Variable	*IDH2*‐R140 *N* = 23	*IDH2*‐R172 *N* = 8	*p* value	*IDH1* *N* = 8	*IDH2* *N* = 31	*p* value
BM blast (%) at diagnosis	68	71	0.8	71	69	0.8
Mid‐induction BM blast (%)	4	10	0.3	4.5	4	0.8
Blast clearance at mid‐induction (%)	68	38	0.2	60	50	0.7
End‐induction BM blast (%)	2	4	0.01*	1.5	3	0.1
Blast clearance at end‐induction (%)	100	75	0.06	87.5	93.1	0.5
CRc (%)	87	75	0.5	75	84	0.6
Median time to clearance (days)	31	37	0.3	33	31	0.9
log_10_ fold reduction in blasts	1.4	1.22	0.21	1.63	1.35	0.23
Rate of blast reduction (log_10_ / day)	0.04	0.025	0.15	0.045	0.04	0.14
Median OS (months)	66.8	NR	0.4	44.4	NR	0.7

*Note*: Comparison of treatment response parameters between patients with *IDH2‐R140*, *IDH2‐R172*, *IDH1*, and *IDH2* mutations. Variables assessed include BM blast percentage at diagnosis, mid‐induction bone marrow (BM) blast percentage, blast clearance at mid‐induction, end‐induction BM blast percentage, blast clearance at end‐induction, composite remission (CRc), which is remission (CR) and complete remission with incomplete hematologic recovery (CRi), median time to clearance, and median overall survival (OS). Values represent percentages or medians, as indicated. *p* values were calculated for the indicated comparisons; ^*^ *p* < 0.05 was considered statistically significant.

Abbreviations: BM, bone marrow; CRc, composite remission; NR, not reached; OS, overall survival.

Our findings align with the report from Salman et al. report suggesting slower blast burden reduction in *IDH2* R172 AML compared with R140 [5]. Although our analysis did not reach statistical significance, likely due to small size, *IDH2 R172* consistently showed trends toward higher bone marrow blast percentages, delayed response, and lower CRc rates. In contrast, *IDH2*‐R140 demonstrated greater and faster blast clearance following intensive induction, as reflected by higher median log_10_ fold reduction and a faster rate of blast decline (log_10_/day). These findings suggest a biologically more responsive phenotype in R140‐mutated AML. This difference may reflect underlying structural differences in the mutant enzyme isoforms, which are known to differentially modulate oncometabolite (R)‐2‐hydroxyglutarate production and cellular differentiation [7]. Importantly, while these kinetic trends did not translate into statistically significant survival differences, early responders (achieving CR/CRi after the first induction) demonstrated a tendency toward longer survival, highlighting the potential prognostic relevance of early cytoreduction.

When comparing *IDH1* and *IDH2*‐mutated AML, we found no significant difference in the magnitude or rate of blast reduction (median log_10_ fold reduction and log_10_/day), or OS, consistent with Middeke et al. findings [8], indicating that prognostic impact may be more mutation site‐specific rather than gene‐specific. Our study has several limitations, including the small sample size in each mutation subgroup and retrospective single‐center design. In addition, treatment regimens were not stratified beyond intensive induction. Finally, we did not have patients who received IDH inhibitors incorporated in their regimens that could impact this phenomenon.

In conclusion, AML patients with *IDH2* R140 mutations tend to achieve more rapid and complete blast cytoreduction following intensive induction compared with *IDH2* R172, while *IDH1*‐mutated patients showed comparable outcomes to the *IDH2* group overall. These results highlight potential biological differences between *IDH* mutation subtypes that may be clinically relevant for response assessment and risk stratification. Larger multicenter analyses are warranted to validate these observations and explore their therapeutic implications.

## Author Contributions

Project design: Mohamad Sheikh Najeeb, Aref Al‐Kali, and Tariq Kewan. Data analysis: Mohamad Sheikh Najeeb. Manuscript writing and review: All authors.

## Funding

The authors have nothing to report.

## Conflicts of Interest

The authors declare no conflicts of interest.

## Supporting information




**Supporting File 1**: jha270204‐sup‐0001‐figureS1.pdf

**Supporting File 2**: jha270204‐sup‐0002‐figureS2.pdf

**Supporting File 3**: jha270204‐sup‐0003‐figureS3.pdf

**Supporting File 4**: jha270204‐sup‐0004‐figureS4.pdf

**Supporting File 5**: jha270204‐sup‐0005‐figureS5.pdf

**Supporting File 6**: jha270204‐sup‐0006‐figureS6.pdf

**Supporting File 7**: jha270204‐sup‐0007‐figureS7.pdf

**Supporting File 8**: jha270204‐sup‐0008‐figureS8.pdf

**Supporting File 9**: jha270204‐sup‐0009‐figureS9.pdf

**Supporting File 10**: jha270204‐sup‐0010‐figureS10.pdf

**Supporting File 11**: jha270204‐sup‐0011‐figureS11.pdf

**Supporting File 12**: jha270204‐sup‐0012‐figureS12.pdf

**Supporting File 13**: jha270204‐sup‐0013‐figureS13.pdf

**Supporting File 14**: jha270204‐sup‐0014‐figureS14.pdf

**Supporting File 15**: jha270204‐sup‐0015‐figureS15.pdf

**Supporting File 16**: jha270204‐sup‐0016‐figureS16.pdf

**Supporting File 17**: jha270204‐sup‐0017‐figureS17.pdf

**Supporting File 18**: jha270204‐sup‐0018‐figureS18.pdf

**Supporting File 19**: jha270204‐sup‐0019‐figureS19.pdf

**Supporting File 20**: jha270204‐sup‐0020‐figureS20.pdf

**Supporting File 21**: jha270204‐sup‐0021‐figureS21.pdf

**Supporting File 22**: jha270204‐sup‐0022‐figureS22.pdf

**Supporting File 23**: jha270204‐sup‐0023‐SuppMat.docx

## Data Availability

For original data, please contact alkali.aref@mayo.edu.
