# Supplementary figures and images for: Blast Clearance Dynamics and Time to Response Across IDH1‐ and IDH2‐Mutated AML
Source: EJHaem. 2025 Dec 15;6(6):e70204. doi: 10.1002/jha2.70204 (PMC12703798; doi:10.1002/jha2.70204)

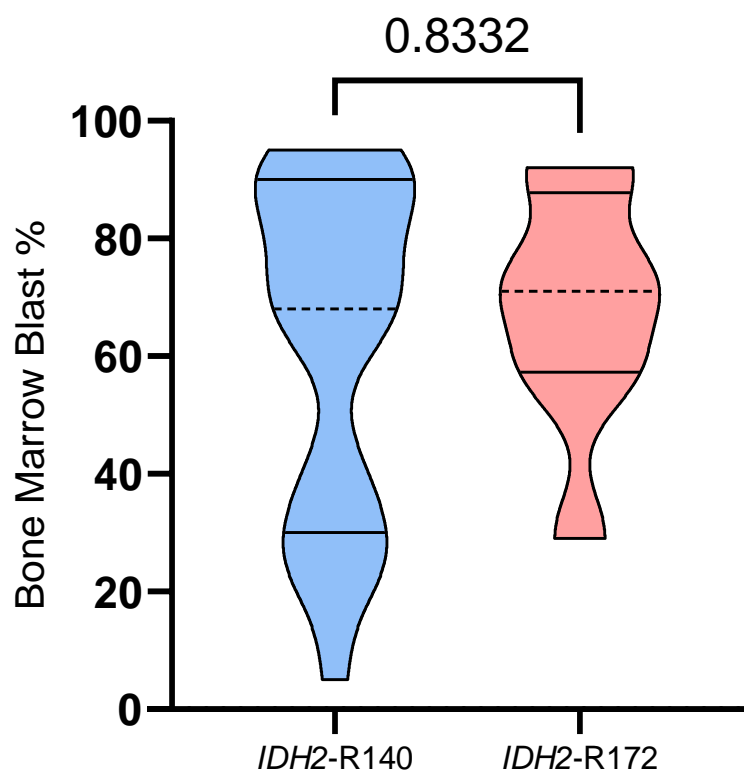

Supplement: Supplementary file 1 — Supporting File 1: jha270204‐sup‐0001‐figureS1.pdf [file JHA2-6-e70204-s004.pdf]

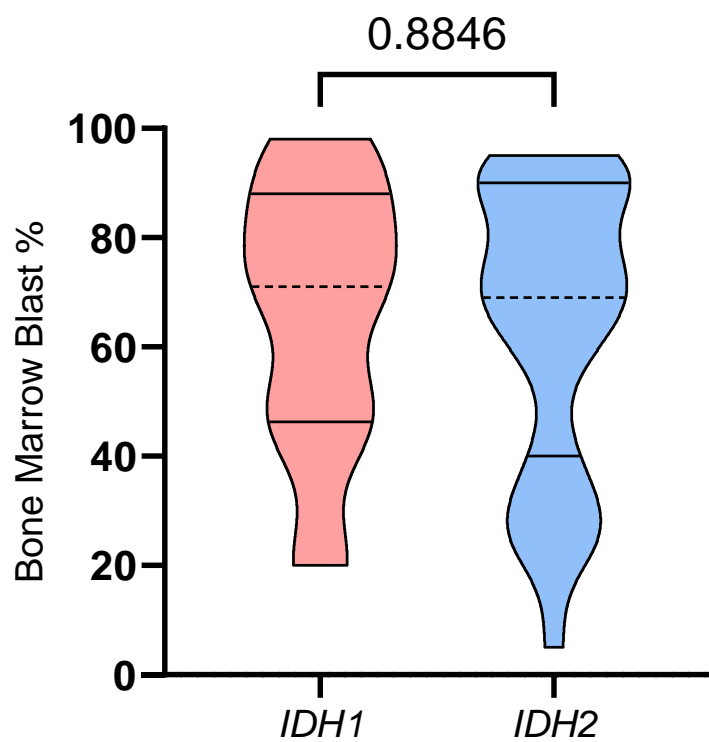

Supplement: Supplementary file 2 — Supporting File 2: jha270204‐sup‐0002‐figureS2.pdf [file JHA2-6-e70204-s003.pdf]

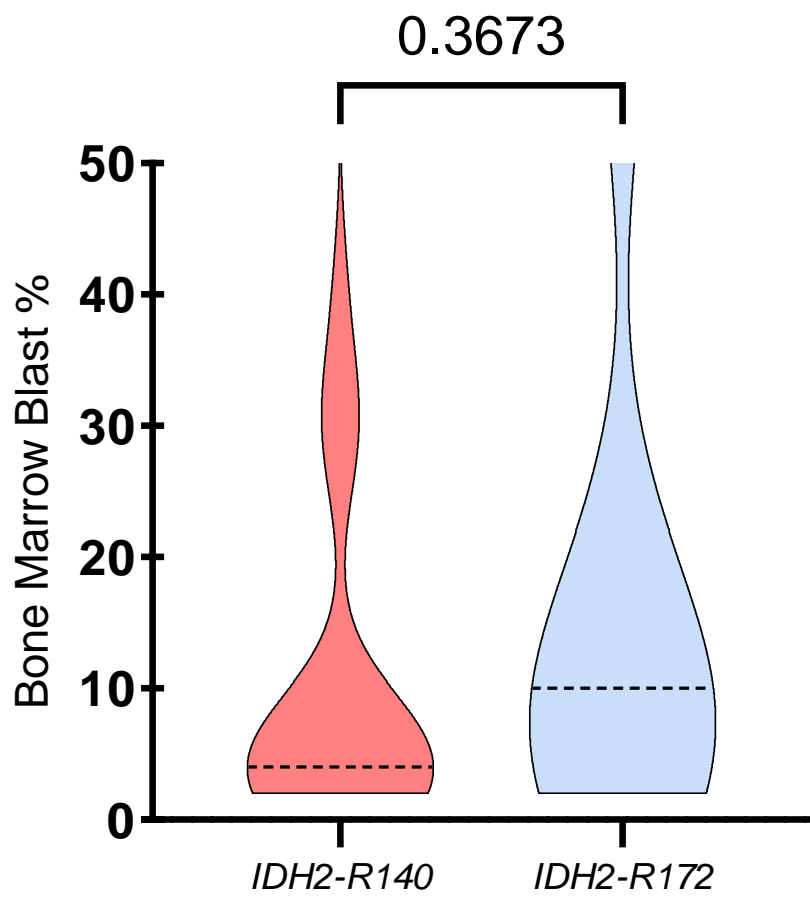

Supplement: Supplementary file 3 — Supporting File 3: jha270204‐sup‐0003‐figureS3.pdf [file JHA2-6-e70204-s021.pdf]

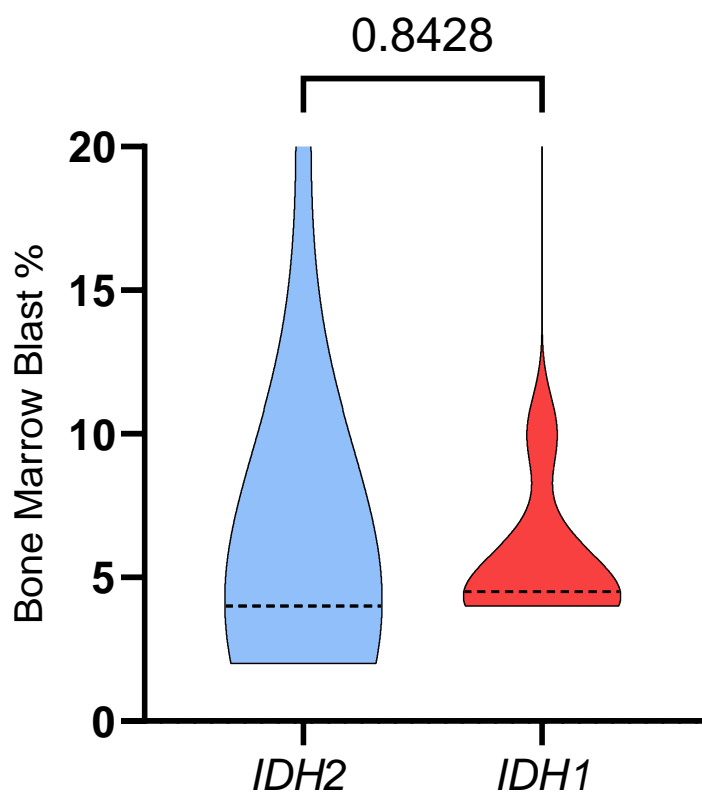

Supplement: Supplementary file 4 — Supporting File 4: jha270204‐sup‐0004‐figureS4.pdf [file JHA2-6-e70204-s001.pdf]

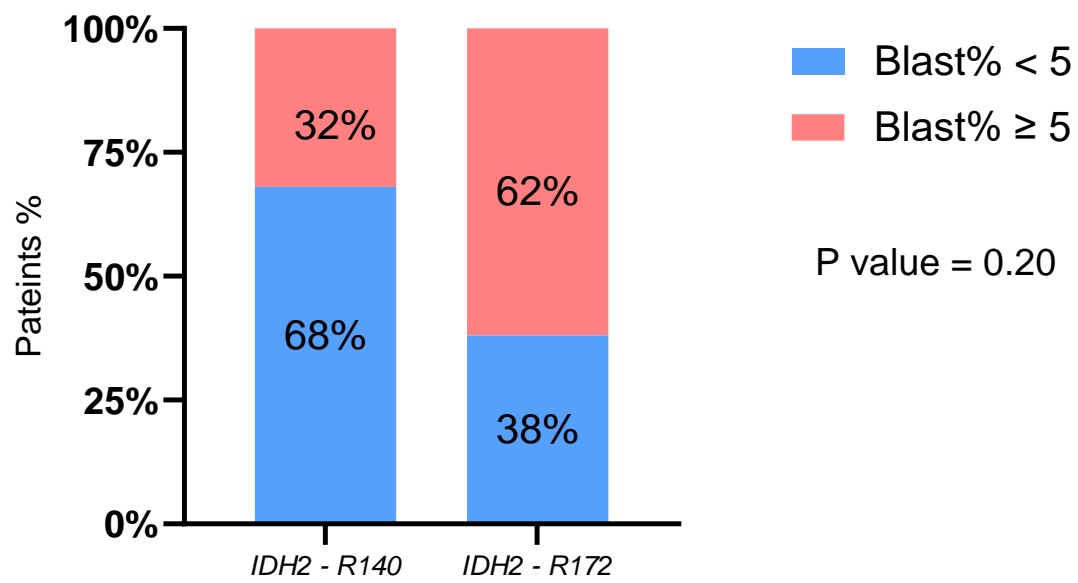

Supplement: Supplementary file 5 — Supporting File 5: jha270204‐sup‐0005‐figureS5.pdf [file JHA2-6-e70204-s009.pdf]

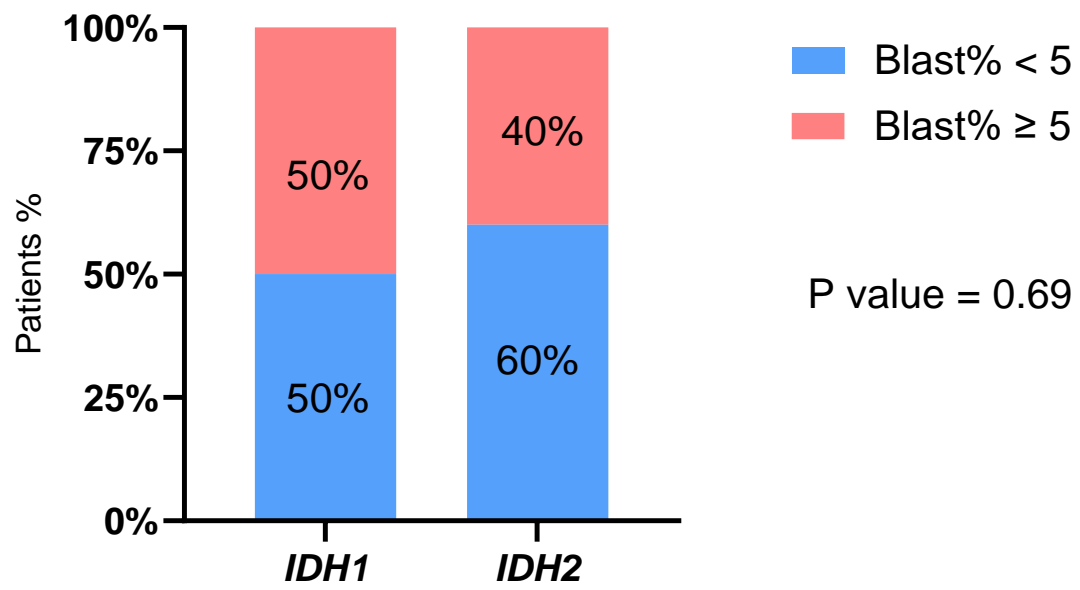

Supplement: Supplementary file 6 — Supporting File 6: jha270204‐sup‐0006‐figureS6.pdf [file JHA2-6-e70204-s013.pdf]

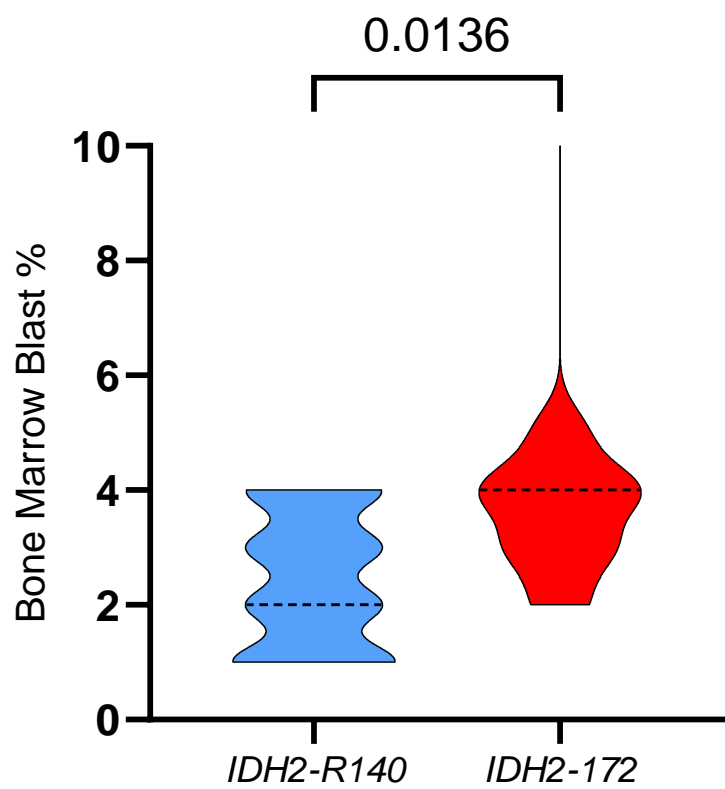

Supplement: Supplementary file 7 — Supporting File 7: jha270204‐sup‐0007‐figureS7.pdf [file JHA2-6-e70204-s020.pdf]

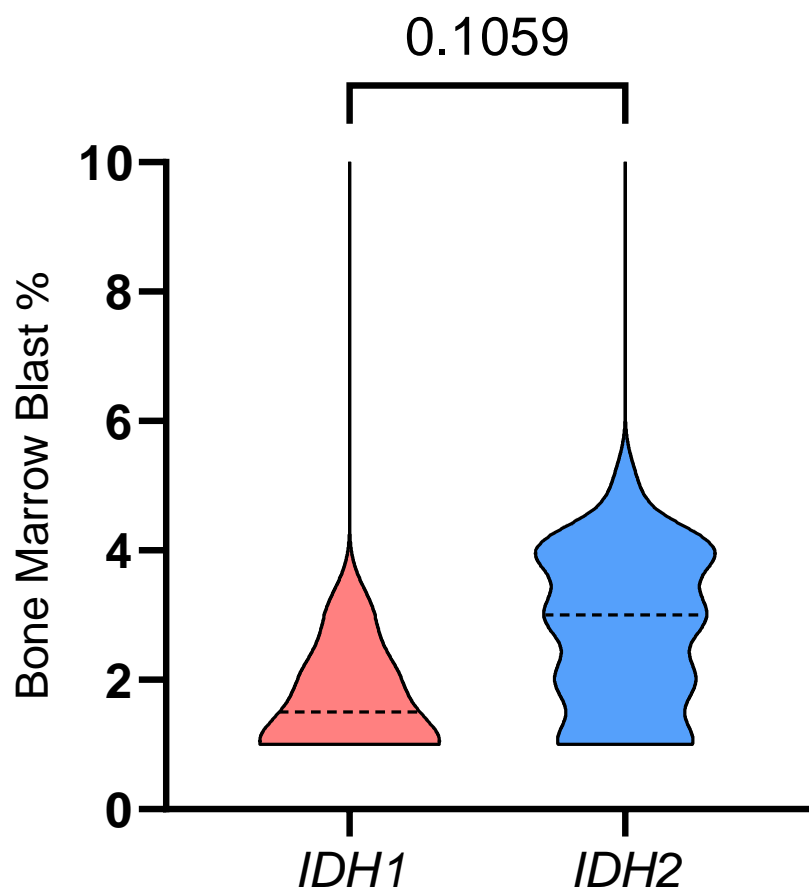

Supplement: Supplementary file 8 — Supporting File 8: jha270204‐sup‐0008‐figureS8.pdf [file JHA2-6-e70204-s014.pdf]

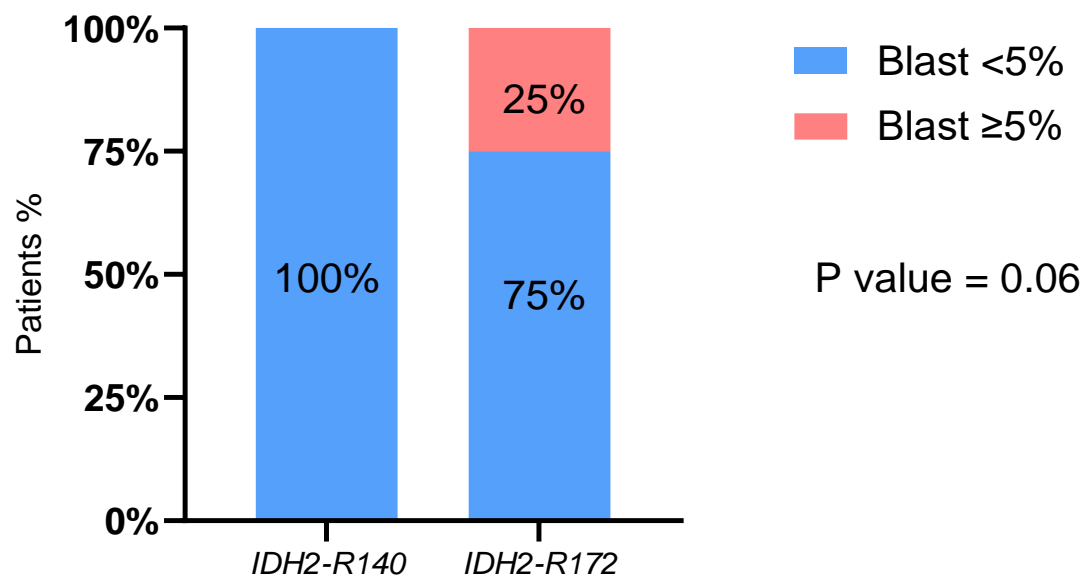

Supplement: Supplementary file 9 — Supporting File 9: jha270204‐sup‐0009‐figureS9.pdf [file JHA2-6-e70204-s006.pdf]

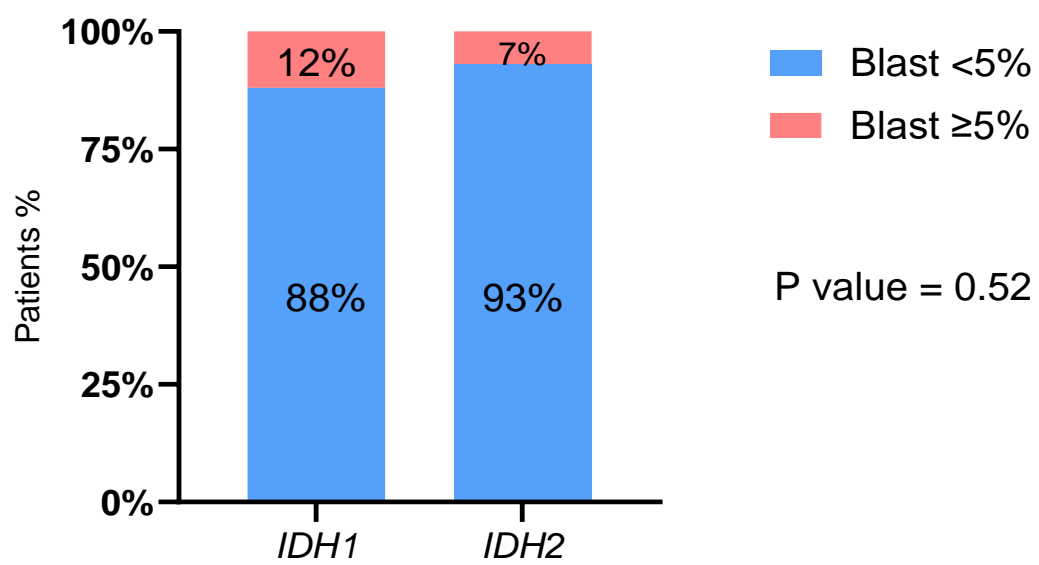

Supplement: Supplementary file 10 — Supporting File 10: jha270204‐sup‐0010‐figureS10.pdf [file JHA2-6-e70204-s012.pdf]

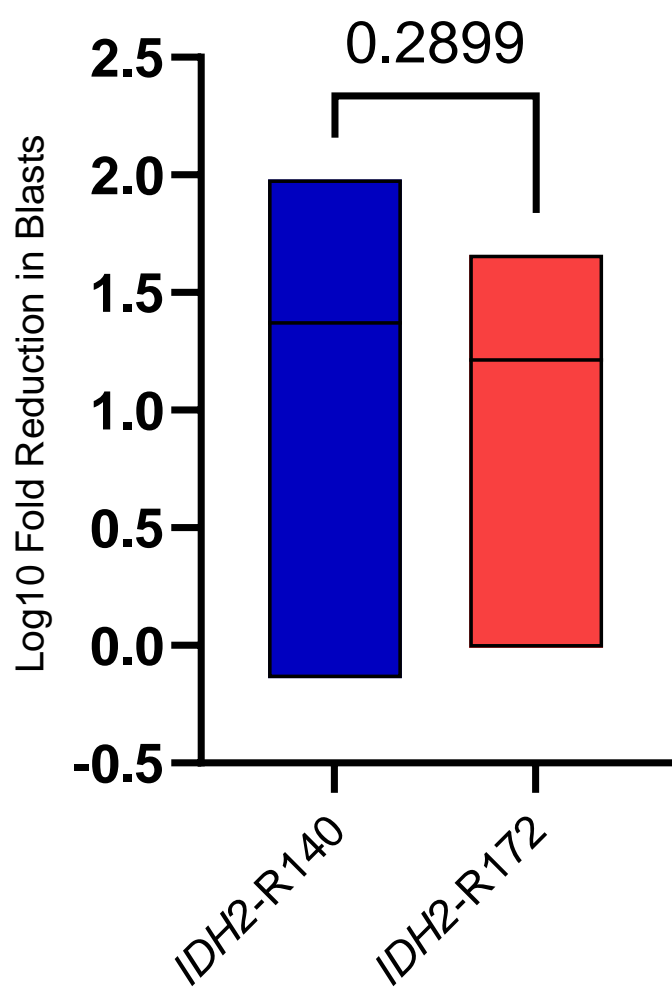

Supplement: Supplementary file 11 — Supporting File 11: jha270204‐sup‐0011‐figureS11.pdf [file JHA2-6-e70204-s022.pdf]

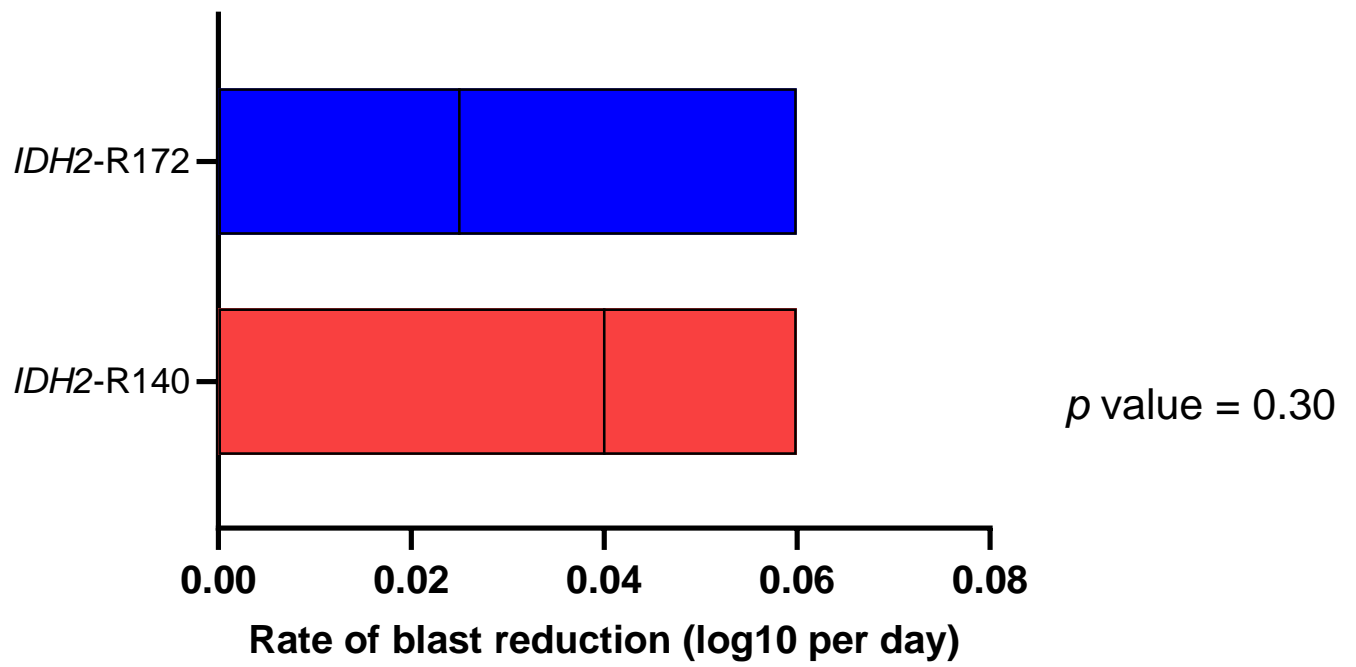

Supplement: Supplementary file 12 — Supporting File 12: jha270204‐sup‐0012‐figureS12.pdf [file JHA2-6-e70204-s005.pdf]

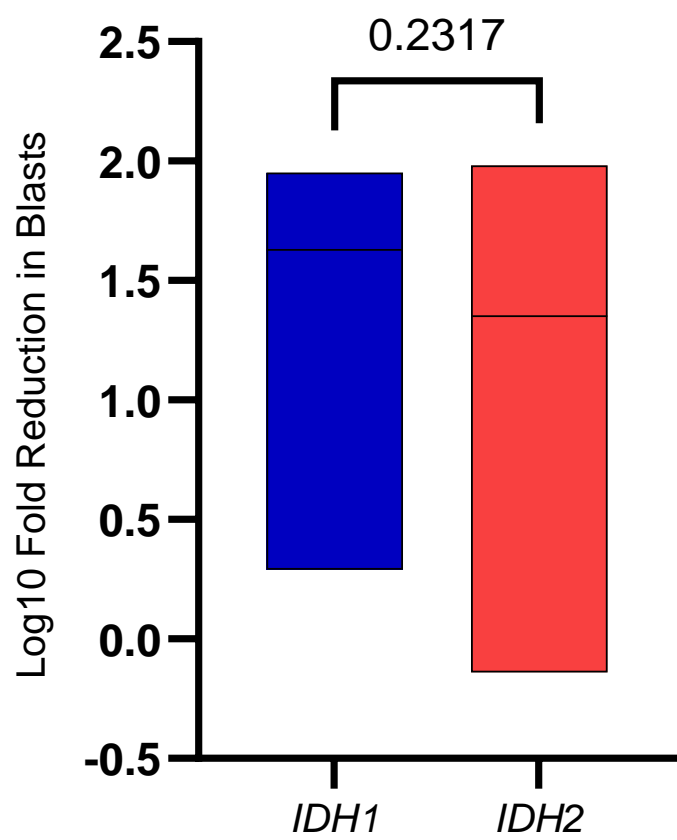

Supplement: Supplementary file 13 — Supporting File 13: jha270204‐sup‐0013‐figureS13.pdf [file JHA2-6-e70204-s018.pdf]

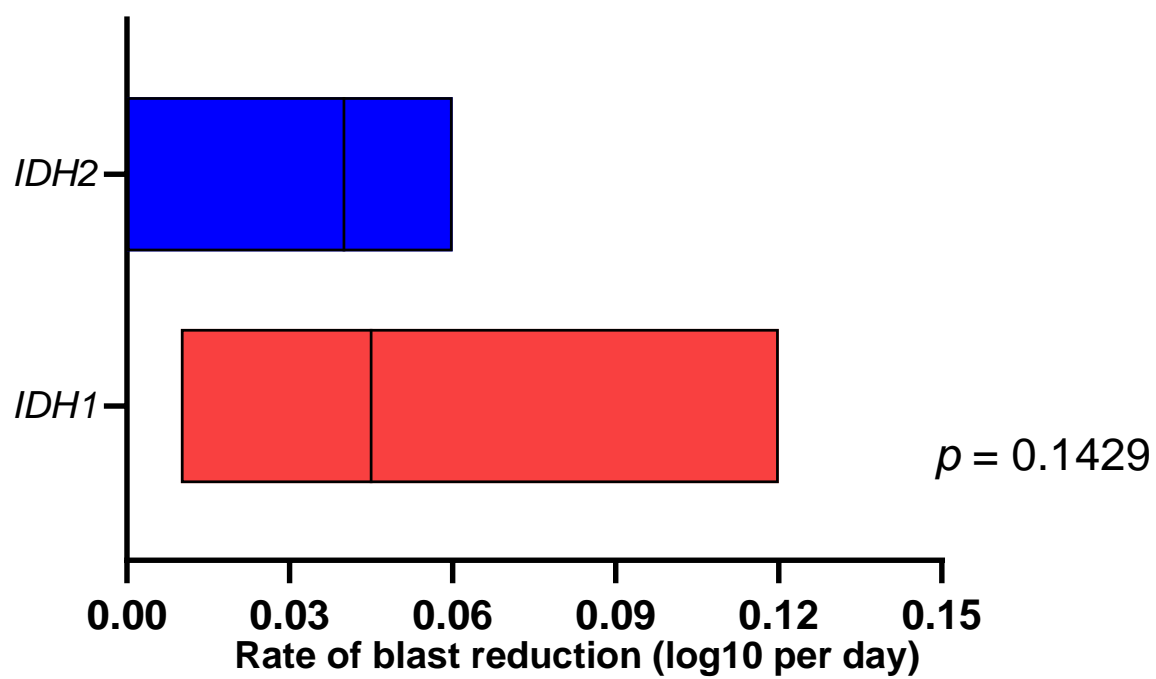

Supplement: Supplementary file 14 — Supporting File 14: jha270204‐sup‐0014‐figureS14.pdf [file JHA2-6-e70204-s017.pdf]

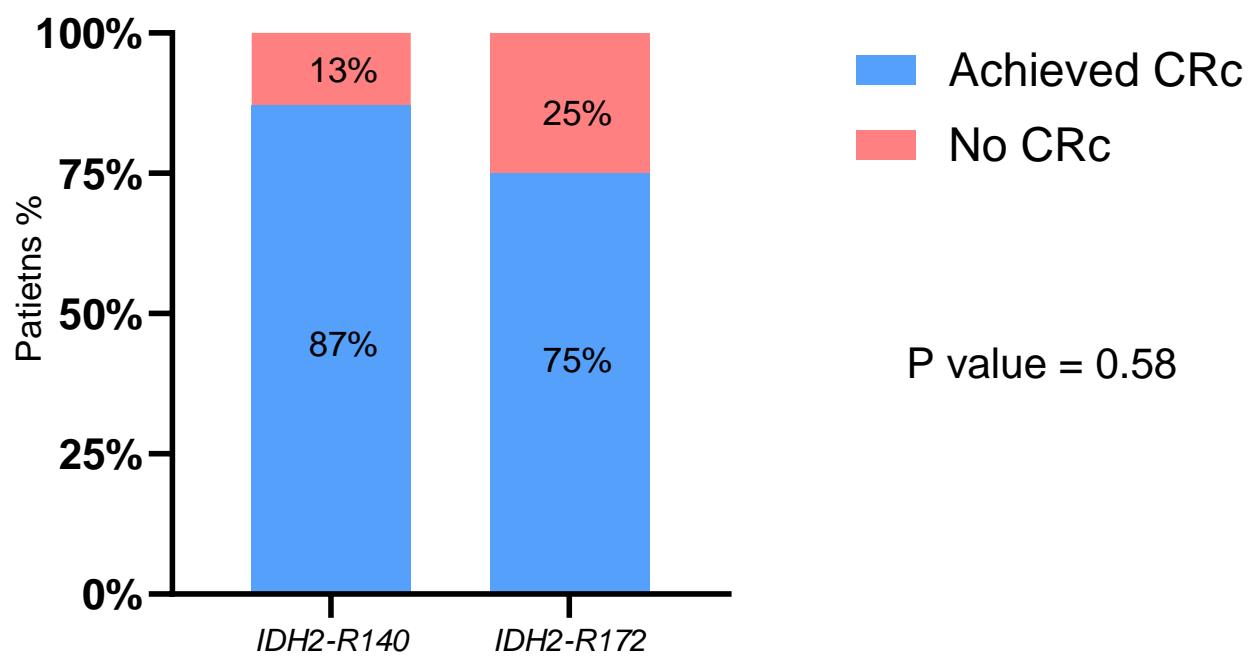

Supplement: Supplementary file 15 — Supporting File 15: jha270204‐sup‐0015‐figureS15.pdf [file JHA2-6-e70204-s023.pdf]

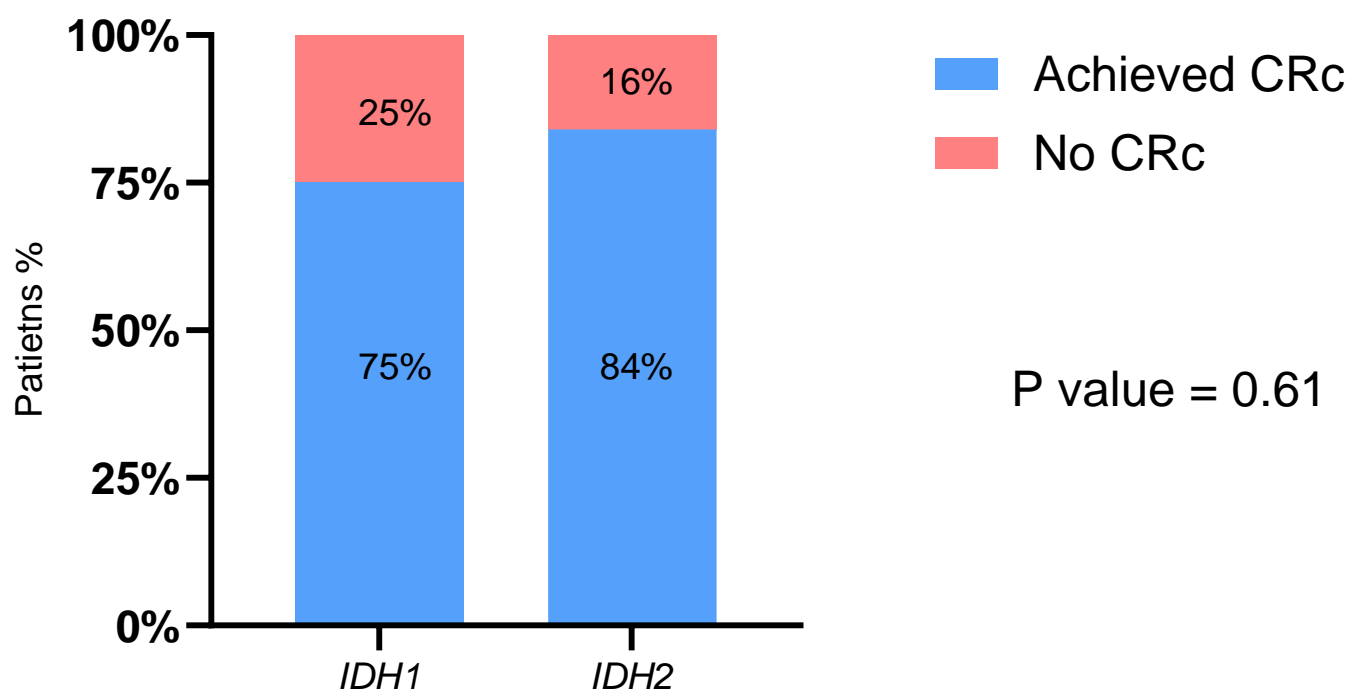

Supplement: Supplementary file 16 — Supporting File 16: jha270204‐sup‐0016‐figureS16.pdf [file JHA2-6-e70204-s008.pdf]

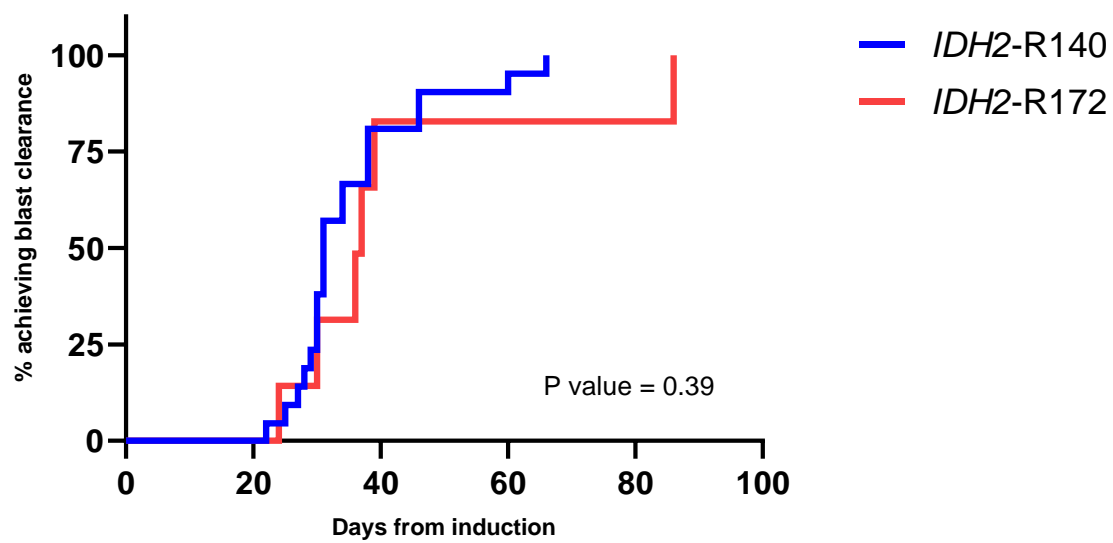

Supplement: Supplementary file 17 — Supporting File 17: jha270204‐sup‐0017‐figureS17.pdf [file JHA2-6-e70204-s010.pdf]

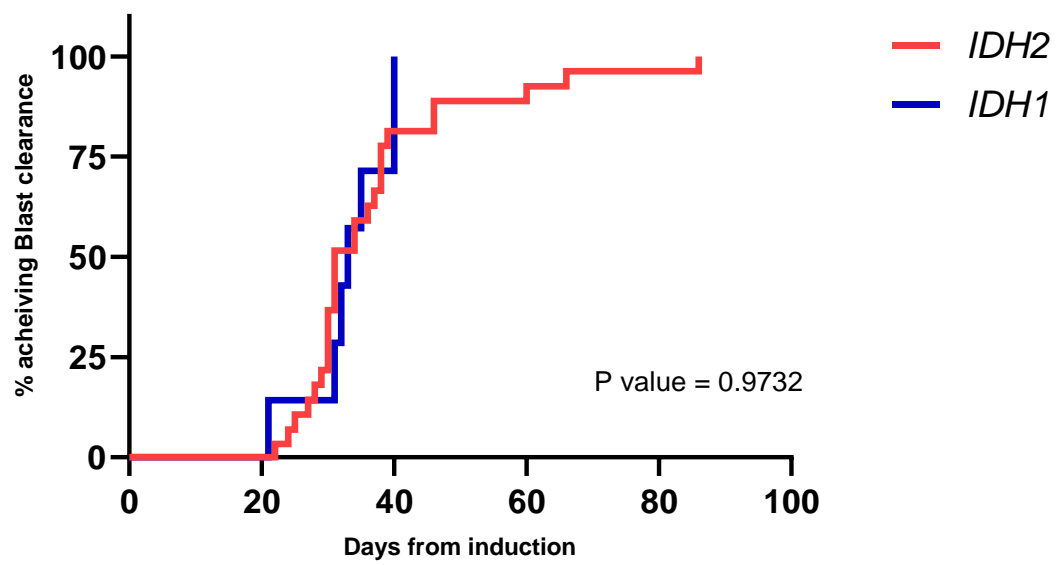

Supplement: Supplementary file 18 — Supporting File 18: jha270204‐sup‐0018‐figureS18.pdf [file JHA2-6-e70204-s002.pdf]

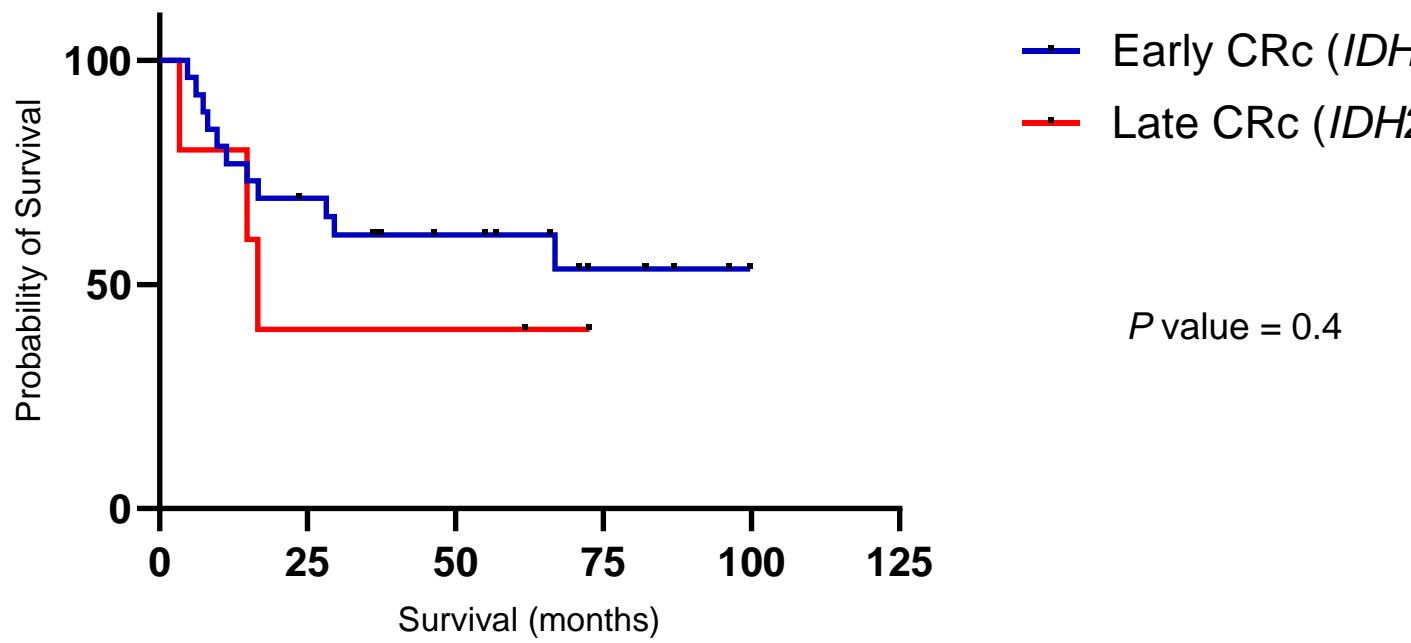

Supplement: Supplementary file 19 — Supporting File 19: jha270204‐sup‐0019‐figureS19.pdf [file JHA2-6-e70204-s015.pdf]

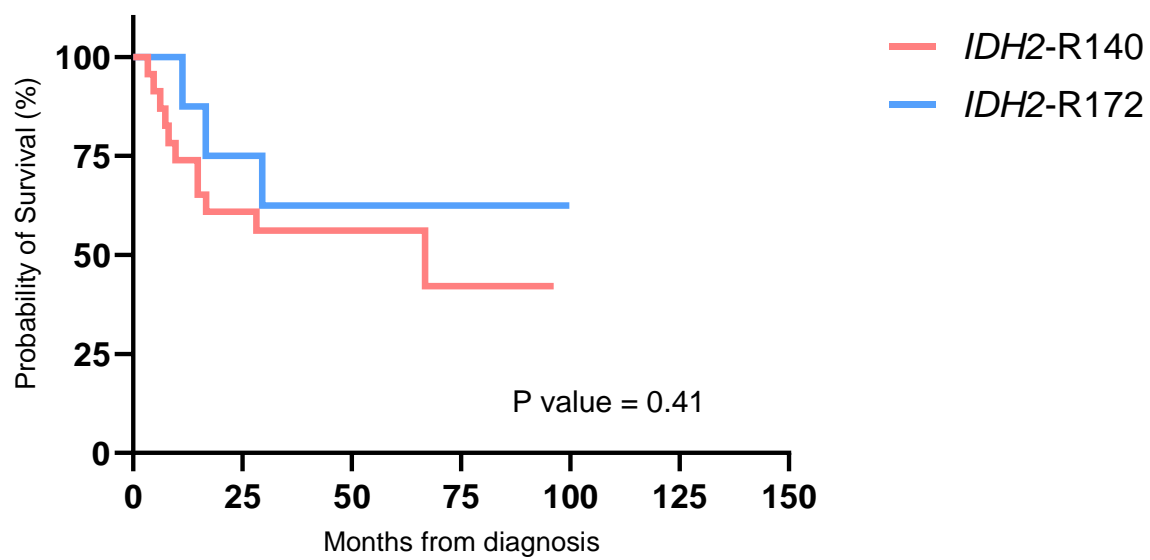

Supplement: Supplementary file 20 — Supporting File 20: jha270204‐sup‐0020‐figureS20.pdf [file JHA2-6-e70204-s007.pdf]

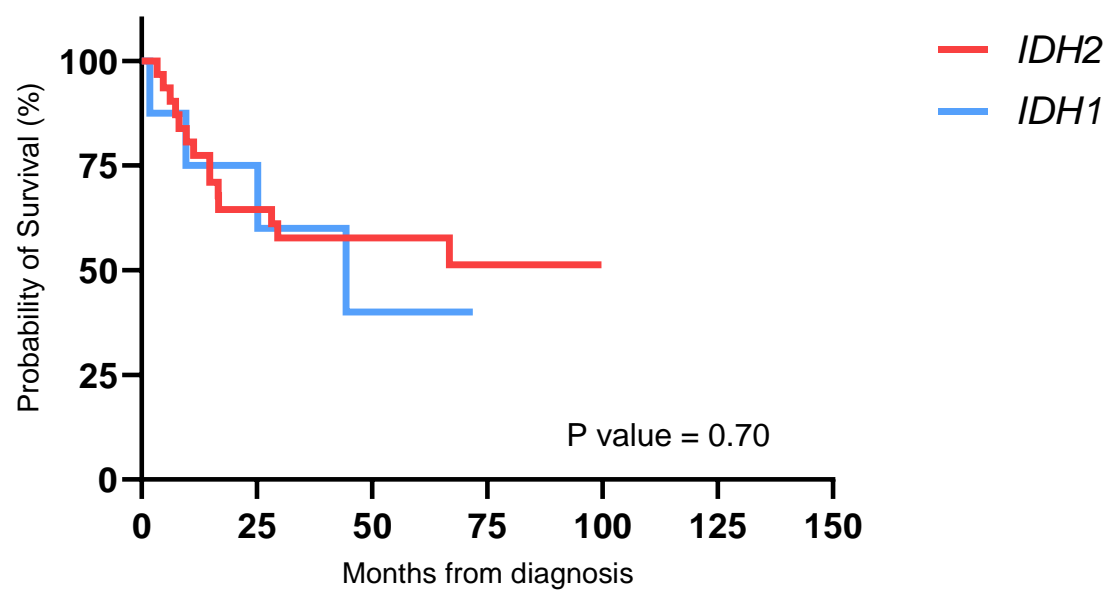

Supplement: Supplementary file 21 — Supporting File 21: jha270204‐sup‐0021‐figureS21.pdf [file JHA2-6-e70204-s011.pdf]

Distribution of ELN 2022 Classification by *IDH2* Subtype

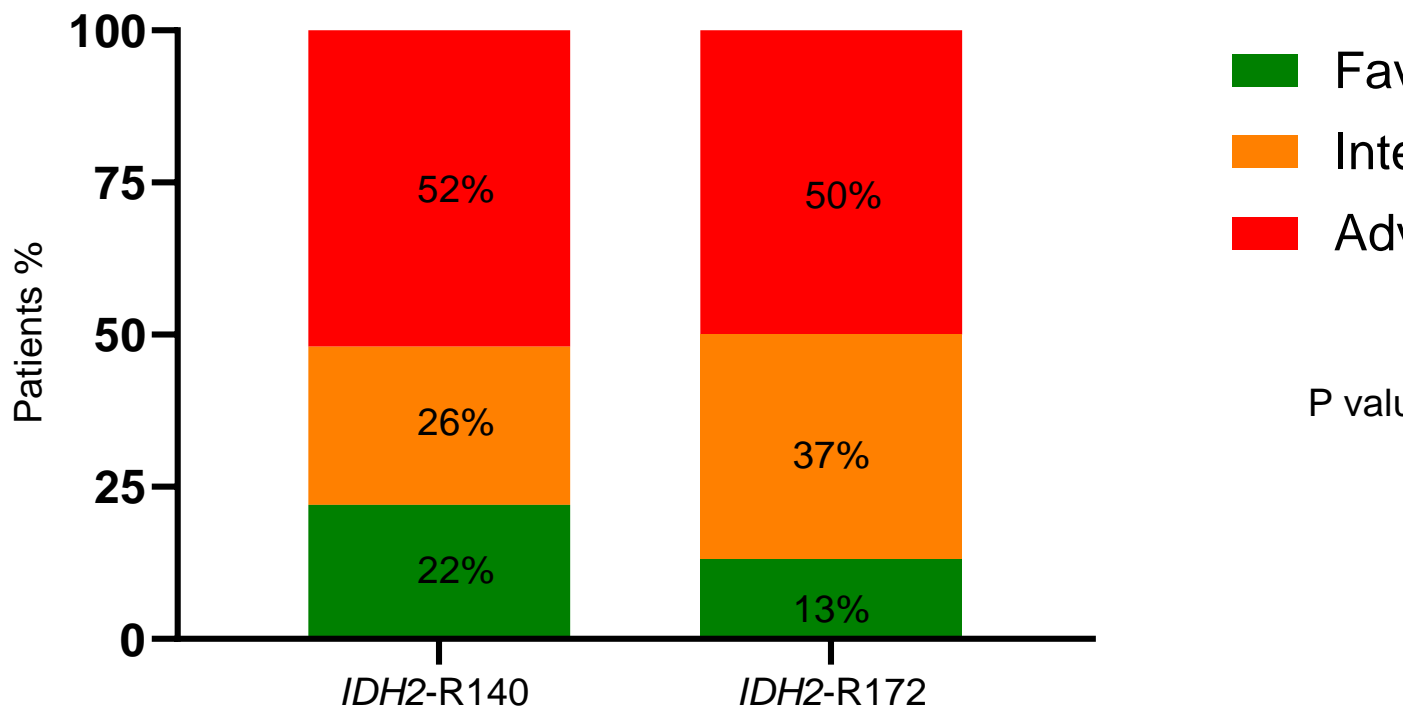

Supplement: Supplementary file 22 — Supporting File 22: jha270204‐sup‐0022‐figureS22.pdf [file JHA2-6-e70204-s016.pdf]
