# Supplementary material for: Blast Clearance Dynamics and Time to Response Across IDH1‐ and IDH2‐Mutated AML
Source: EJHaem. 2025 Dec 15;6(6):e70204. doi: 10.1002/jha2.70204 (PMC12703798; doi:10.1002/jha2.70204)
Supplement: Supplementary file 23 — Supporting File 23: jha270204‐sup‐0023‐SuppMat.docx [file JHA2-6-e70204-s019.docx]

**Supplemental Methods:**

**Mutation analysis and Next Generation Sequencing**

Next-generation sequencing (NGS) analysis was conducted on peripheral blood or bone marrow specimens, utilizing a comprehensive gene list (OncoHeme, Mayo Clinic) encompassing 35-47 genes recurrently mutated in myeloid neoplasms across evolving versions of the assay. This panel currently included *EZH2* (NM_004456.4) exons 3-21, as well as other genes associated with myeloid neoplasms: *ANKRD26*, *ASXL1, BCOR, BCORL1, BRAF, CALR, CBL, CEBPA, CSF3R, DDX41, DNMT3A, ELANE, ETNK1, ETV6, EZH2, FLT3, GATA1, GATA2, IDH1, IDH2, JAK2, KDM6A, KIT, KRAS, MPL, NF1, NPM1, NRAS, PHF6, PPM1D, PTPN11, RAD21, RUNX1, SETBP1, SH2B3, SF3B1, SMC3, SRSF2, STAG2, STAT3, TERT, TET2, TP53, U2AF1, UBA1, WT1,* and *ZRSR2*. DNA underwent target enrichment with a custom hybridization-capture reagent (SureSelectXT, Agilent, Santa Clara, CA) and sequencing on the MiSeq or HiSeq platforms (Illumina, San Diego, CA) at the Mayo Clinic Clinical Genome Sequencing Laboratory. The sequencing data underwent processing through a custom bioinformatics pipeline (Mayo NGS Workbench), using CLC Bio Genomics Server v6.0 (Qiagen, Redwood City, CA) for alignment and variant calling. The limit of detection of the NGS assay is 5% with a minimum 250X depth of coverage. Over 95% of tested regions had >1000X depth of coverage in the clinical assay. Genetic variants were curated and annotated in the Mayo Clinic Molecular Hematopathology Laboratory following the American College of Medical Genetics and Genomics (ACMG) five-tier system. The NGS panel has accuracy >99%; reproducibility 100% (both intra- and interassay). Its sensitivity ranges from 2-5% variant allele fraction, with a minimum depth coverage of 250X for single base substitutions and insertion/deletion event.

Supplemental Table1.: Baseline characteristics of patients with *IDH*-mutated acute myeloid leukemia (AML).

| Patient # | Gender | Age (y) | ELN 2022 classification (Favorable=0, Intermediate=1, Adverse=2) | Hgb (g/dL) | WBC (×10⁹/L) | Platelets (×10⁹/L) | Abnormal cytogenetics | *IDH* subtype | Co-mutations |
| --- | --- | --- | --- | --- | --- | --- | --- | --- | --- |
| N408 | F | 59 | 2 | 10.7 | 1.5 | 16 | — | *IDH2*-R140 | *BCOR, DNMT3A, RUNX1, Multiple RUNX1, SH2B3* |
| N11 | F | 68 | 0 | 8 | 2.7 | 48 | — | *IDH2*-R140 | *CEBPA, DNMT3A, NPM1* |
| N36 | M | 71 | 0 | 8.1 | 4 | 52 | — | *IDH2*-R140 | *CEBPA, Multiple CEBPA, SRSF2* |
| N47 | M | 65 | 0 | 11.7 | 2.2 | 120 | — | *IDH2*-R140 | *NPM1, ZRSR2* |
| N94 | M | 66 | 2 | 7.5 | 103.5 | 80 | 45,XY,-7[20] | *IDH2*-R140 | *DNMT3A, KRAS, SRSF2* |
| N140 | F | 71 | 0 | 8.6 | 0.9 | 122 | — | *IDH2*-R140 | *NPM1, TET2* |
| N240 | F | 65 | 0 | 9.1 | 10.6 | 64 | — | *IDH2*-R140 | *NPM1, SRSF2* |
| N304 | F | 56 | 1 | 5.6 | 93.6 | 27 | — | *IDH2*-R140 | *FLT3_ITD, NPM1* |
| N345 | M | 47 | 1 | 7.2 | 31 | 163 | — | *IDH2*-R140 | *DNMT3A, FLT3_ITD, Multiple FLT3, NPM1* |
| N362 | F | 59 | 1 | 7.7 | 90.1 | 11 | — | *IDH2*-R140 | *FLT3_ITD, Multiple FLT3, NPM1* |
| N146 | M | 58 | 1 | 11.4 | 6.6 | 139 | — | *IDH2*-R140 | *ASXL1, BCOR, FLT3_ITD, RUNX1, STAG2* |
| N176 | F | 51 | 1 | 6.7 | 12.4 | 12 | — | *IDH2*-R140 | *FLT3_ITD, Multiple FLT3, NPM1, PHF6,* |
| N354 | M | 61 | 2 | 8.2 | 1.6 | 64 | 45,XY,-7[3]/46,XY[17] | *IDH2*-R140 | *ASXL1, PHF6, RUNX1, U2AF1* |
| N468 | M | 65 | 2 | 8.2 | 74.4 | 97 |  | *IDH2*-R140 | *FLT3_ITD, Multiple FLT3, NRAS, RUNX1, WT1* |
| N222 | M | 64 | 2 | 9 | 32.1 | 74 |  | *IDH2*-R140 | *ASXL1, BCOR, KRAS, STAG2* |
| N46 | F | 72 | 2 | 7.5 | 2.1 | 48 | 51-55,XX,+1,+2,+18[cp2]/46,XX[18] | *IDH2*-R140 | *ASXL1, CEBPA, EZH2,* |
| N221 | M | 61 | 1 | 7.3 | 3.2 | 20 | — | *IDH2*-R140 | *FLT3_ITD, NPM1, SRSF2* |
| N373 | M | 73 | 2 | 9.4 | 9.6 | 50 | — | *IDH2*-R140 | *ASXL1, NRAS, RUNX1, SRSF2, STAG2* |
| N101 | M | 67 | 2 | 10.5 | 2.6 | 59 | — | *IDH2*-R140 | *ASXL1, SRSF2* |
| N56 | M | 68 | 2 | 10.6 | 1.4 | 73 | 46,XY,der(7)t(7;15)(q22;q21)[5]/46,XY[15] | *IDH2*-R140 | *DNMT3A, JAK2_V617F, NRAS, RUNX1, SRSF2, TP53* |
| N5 | M | 68 | 2 | 10.2 | 2 | 145 | 47,XY,+11[1]/46,XY[19] | *IDH2*-R140 | *ASXL1, SRSF2* |
| N195 | M | 67 | 2 | 7.6 | 1.5 | 125 | 46,XY,del(12)(p11.2p13)[9]/46,XY[11] | *IDH2*-R140 | *BCOR, DNMT3A, STAG2* |
| N17 | M | 73 | 2 | 7.8 | 204.4 | 21 | — | *IDH2*-R140 | *FLT3_ITD, GATA2, SRSF2* |
| N33 | F | 41 | 0 | 7.8 | 9.2 | 69 | 46,XX,del(9)(q13q22)[15]/47,sl,+4[3]/46,XX[2] | *IDH2*-R172 | *NPM1* |
| N15 | F | 62 | 2 | 8.5 | 0.7 | 125 | — | *IDH2*-R172 | *DNMT3A, RUNX1* |
| N24 | F | 37 | 1 | 8.2 | 2.6 | 52 | 46,XX,add(7)(q11.2)[20] | *IDH2*-R172 | *EZH2* |
| N73 | F | 52 | 1 | 10.4 | 1.7 | 122 | 46,XX,idic(7)(q11.2)[7]/46,XX[13] | *IDH2*-R172 | *DNMT3A* |
| N135 | M | 26 | 1 | 6.3 | 48.6 | 15 | — | *IDH2*-R172 | *FLT3_ITD, PTPN11, WT1* |
| N105 | M | 59 | 2 | 9.9 | 1.2 | 179 | — | *IDH2*-R172 | *JAK2_V617F, RUNX1* |
| N137 | M | 66 | 2 | 6.4 | 1.7 | 100 | — | *IDH2*-R172 | *BCOR, DNMT3A, NRAS* |
| N331 | F | 62 | 2 | 8.8 | 2.4 | 240 | — | *IDH2*-R172 | *ASXL1, DNMT3A* |
| N22 | M | 61 | 1 | 10.4 | 1 | 144 | 46,XY[20} | *IDH1*-R132 | *DNMT3A* |
| N83 | M | 56 | 2 | 10.5 | 5.4 | 156 | 46,XY[20] | *IDH1*-R132 | *DNMT3A, SRSF2* |
| N152 | M | 48 | 2 | _ | _ | _ | 46,XY,t(8;21)(q13;q21),t(9;22)(q34;q11.2)[9]/47,sl,+9[8]/47, sl,+8[3] | *IDH1*-R132 | *_* |
| N173 | M | 72 | 1 | 10.4 | 53.4 | 21 | 47,XY,+8[7]/46,XY[13]. | *IDH1*-R132 | *DNMT3A, FLT3_TKD, Multiple FLT3, NRAS, Multiple NRAS, PHF6,* |
| N224 | M | 63 | 2 | 9.2 | 1 | 149 | 46,XY[20] | *IDH1*-R132 | *SF3B1* |
| N239 | F | 50 | 1 | 9.9 | 2.3 | 31 | 47,XX,+8[3]/46,XX[17] | *IDH1*-R132 | *PHF6, RAD21* |
| N457 | F | 39 | 0 | 10.9 | 2.4 | 14 | 47,XX,+8[12]/48,sl,+9[7]/46,XX[1] | *IDH1*-R132 | *CEBPA, RUNX1,* |
| N502 | F | 40 | 2 | 9 | 1.9 | 76 | 46,XX[20] | *IDH1*-R132 | *ASXL1, DNMT3A, STAG2* |

**Supplemental Table1. Baseline characteristics of patients with *IDH*-mutated acute myeloid leukemia (AML).**Clinical, cytogenetic, and molecular features of patients harboring *IDH1* (R132) or *IDH2* (R140, R172) mutations. ELN 2022 risk classification: 0 = favorable, 1 = intermediate, 2 = adverse. Cytogenetic abnormalities are reported using ISCN 2020 nomenclature. Gene symbols follow HUGO guidelines; only co-mutations with a pathogenic significance are listed. CBC values represent laboratory results at diagnosis.

**Supplemental Figures:**

**Supplemental Figure 1. Bone marrow blast percentages at diagnosis in patients with *IDH2-R140* and *IDH2-R172* mutations.** Violin plots depict the distribution of bone marrow blast percentages at diagnosis. The dashed line indicates the median, and solid lines indicate the interquartile range. Patients with *IDH2-R172* mutations (n = 8; median, 71.0%) and *IDH2-R140* mutations (n = 23; median, 68.0%) showed no significant difference (*p* = 0.83, two-tailed Mann–Whitney U test).

**Supplemental Figure** **2. Bone marrow blast percentages at diagnosis in patients with *IDH1* and *IDH2* mutations.** Violin plots depict the distribution of bone marrow blast percentages at diagnosis. The dashed line indicates the median, and solid lines indicate the interquartile range. Patients with IDH1 mutations (n = 8; median, 71.0%) and IDH2 mutations (n = 31; median, 69.0%) showed no significant difference (p = 0.88, two-tailed Mann–Whitney U test).

**Supplemental Figure** **3.** **Mid-cycle bone marrow blast percentages in AML patients with *IDH2-R140* and *IDH2-R172* mutations.** Violin plots depict the distribution of bone marrow blast percentages at mid-cycle induction chemotherapy. Horizontal dashed lines indicate the median. Patients with *IDH2-R172* mutations (n = 8; median, 10%) had higher blast percentages than those with *IDH2-R140* mutations (n = 22; median, 4%), although the difference was not statistically significant (*p* = 0.37, two-tailed Mann–Whitney U test).

**Supplemental Figure** **4.** **Mid-cycle bone marrow blast percentages in AML patients with *IDH1* and *IDH2* mutations.**

Violin plots depict the distribution of bone marrow blast percentages at mid-cycle induction chemotherapy. Horizontal dashed lines indicate group median. Patients with *IDH2* mutations (n = 30; median, 4%) and *IDH1* mutations (n = 8; median, 4.5%) showed no significant difference (*p* = 0.84, two-tailed Mann–Whitney U test).

**Supplemental Figure** **5.** **Comparison of bone marrow blast percentages at mid-cycle between *IDH2-R140* and *IDH2-R172* mutated AML**. Among patients with *IDH2-R140* (n=22), 68% achieved <5% blasts while 32% had ≥5% blasts, compared with 38% and 62%, respectively, in those with *IDH2-R172* (n=8). The difference between groups did not reach statistical significance (*p* = 0.21, Fisher’s exact test). Bars represent percentages; absolute patient numbers are provided in the legend.

**Supplemental Figure 6. Comparison of mid-cycle bone marrow blast percentages in IDH1 versus IDH2 mutated AML**. At mid-cycle, 50% of *IDH1* patients (n=8) and 60% of IDH2 patients (n=30) achieved <5% blasts, while 50% and 40%, respectively, had ≥5% blasts. No statistically significant difference was observed between the groups (p = 0.69, Fisher’s exact test; odds ratio 0.67, 95% CI 0.17–2.68). Bars represent percentages; absolute patient numbers are provided in the legend.

**Supplemental Figure** **7.** **End-cycle bone marrow blast percentages in AML patients with *IDH2-R140* and *IDH2-R172* mutations.** Violin plots depict the distribution of bone marrow blast percentages at the end of induction chemotherapy. Horizontal dashed lines represent group medians. Patients with *IDH2-R172* mutations (n = 8; median, 4%) had significantly higher blast percentages compared with those with *IDH2-R140* mutations (n = 21; median, 2%; *p* = 0.0136, two-tailed Mann–Whitney U test).

**Supplemental Figure** **8.** **End-cycle bone marrow blast percentages in AML patients with *IDH1* and *IDH2* mutations.** Violin plots depict the distribution of bone marrow blast percentages at the end of induction chemotherapy. Horizontal dashed lines indicate group medians. Patients with *IDH1* (n=8; median 1.5%) and *IDH2* (n=30; median 3%) had comparable blast percentages, with no statistically significant difference observed (*p* = 0.1059, two-tailed Mann–Whitney U test).

**Supplemental Figure** **9. Bone marrow blast percentage at the end of induction in patients with *IDH2-R140* versus *IDH2-R172* mutations.** All patients with *IDH2-R140* (n=21) achieved <5% blasts, whereas only 75% of those with *IDH2-R172* (n=8) had <5% blasts (25% ≥5%). This difference did not reach statistical significance (*p* = 0.06, Fisher’s exact test). Bars represent percentages; absolute patient numbers are provided in the legend.

**Supplemental Figure** **10. Proportion of patients achieving bone marrow blast clearance by IDH mutation subtype.**

Among patients with *IDH1* (n=8), 88% had <5% blasts at follow-up compared with 93% of those with *IDH2* (n=29). The difference was not statistically significant (*p* = 0.52, Fisher’s exact test). Bars represent percentages; absolute patient numbers are provided in the legend.

**Supplemental Figure 11. Log₁₀ Fold Reduction in Bone-Marrow Blasts by *IDH2* Mutation Subtype**

Bar plot comparing the magnitude of blast reduction from diagnosis to end-of-induction between *IDH2-R140 (n = 23)* and *IDH2-R172 (n =8)* AML patients. Bars represent the range of *log₁₀* (blast % at diagnosis / blast % at end-induction), and the solid black line indicates the median. Patients who changed regimen or discontinued induction early were analyzed using their last on-therapy marrow for end-cycle values.
Median log₁₀ fold reduction was 1.37 for R140 and 1.21 for R172 (*p* = 0.2899, Mann–Whitney U test), indicating a trend toward greater blast clearance in R140.

**Supplemental Figure 12. Rate of Blast Fold Reduction by *IDH2* Mutation Subtype**Bar plot comparing the rate of blast clearance between *IDH2-R140* (*n* = 23) and *IDH2-R172* (*n* = 8) AML patients. Each bar represents the range of the *log₁₀*-based rate of blast reduction per day, calculated as *log₁₀* (blast % at diagnosis / blast % at end-induction) divided by the number of days from diagnosis to response assessment. Patients who changed regimen or discontinued induction early due to non-response (*n* = 2, both R172) were analyzed using an intention-to-treat (ITT) approach, in which their *log₁₀* reduction was set to 0 to reflect lack of clearance under the original regimen. Solid black lines indicate the median values. Median rates of blast reduction were 0.040 log₁₀/day for R140 and 0.025 log₁₀/day for R172 (*p* = 0.30, Mann–Whitney U test), showing a trend toward faster blast clearance in R140.

**Supplemental Figure 13. Log₁₀ Fold Reduction in Bone-Marrow Blasts by *IDH1* vs *IDH2* Mutation Subtype**
Bar plot comparing the magnitude of blast reduction from diagnosis to end-of-induction between *IDH1*-mutated (n = 8) and *IDH2*-mutated (n = 31) AML patients. Bars represent the range of log₁₀ (blast % at diagnosis / blast % at end-induction), and the solid black line indicates the median. Patients who changed regimen or discontinued induction early were analyzed using their last on-therapy marrow for end-cycle values. Median log₁₀ fold reduction was 1.63 for *IDH1* and 1.35 for *IDH2* (*p* = 0.2317, Mann–Whitney U test), indicating comparable magnitude of blast clearance between the two subgroups.

**Supplemental Figure 14. Rate of Blast Fold Reduction by *IDH* Mutation Subtype**
Bar plot comparing the rate of blast clearance between *IDH1*-mutated (n = 8) and *IDH2*-mutated (n = 31) AML patients. Each bar represents the range of the log₁₀-based rate of blast reduction per day, calculated as log₁₀ (blast % at diagnosis / blast % at end-induction) divided by the number of days from diagnosis to response assessment. Patients who changed regimen or discontinued induction early were analyzed using an intention-to-treat approach, in which their log₁₀ reduction was set to 0 to reflect lack of clearance under the original regimen. Solid black lines indicate median values. Median rates of blast reduction were 0.045 log₁₀/day for *IDH1* and 0.040 log₁₀/day for *IDH2* (*p* = 0.1429, Mann–Whitney U test), indicating comparable magnitude of blast clearance between the two subgroups.

**Supplemental Figure 15. Rates of Composed Complete Remission (CRc) by IDH2 mutation subtype.**

Among patients with *IDH2-R140* (n=23), 87% achieved CR/CRi compared with 75% of those with *IDH2-R172* (n=8). Differences were not statistically significant (*p* = 0.58, Fisher’s exact test). Bars represent percentages, with absolute patient numbers indicated in the legend.

**Supplemental Figure** **16. Rates of Composed Complete Remission (CRc) among patients with *IDH1* and *IDH2* mutations.** Among patients with *IDH1* (n=8), 75% achieved CR/CRi compared with 84% of those with *IDH2* (n=31). The difference between groups was not statistically significant (*p* = 0.61, Fisher’s exact test). Bars represent percentages; absolute patient numbers are provided in the legend.

**Supplemental Figure 17.** **Comparison of time to blast clearance between *IDH2* R140– and *IDH2* R172–mutated AML.** *Kaplan–Meier* curves depict the proportion of patients achieving blast clearance following induction therapy, stratified by IDH2 mutation subtype (R140 vs. R172). No statistically significant difference in blast clearance kinetics was observed between the two groups (log-rank P = 0.396). The median time to blast clearance was 31 days for *IDH2* R140 and 37 days for *IDH2* R172. Vertical axis shows the percentage of patients achieving blast clearance; horizontal axis represents days from induction start.

**Supplemental Figure 18.** ***Time to blast clearance in AML with IDH1 vs. IDH2 mutations.***Kaplan–Meier curves show the proportion of patients achieving blast clearance from the start of induction therapy, stratified by IDH mutation type (*IDH1* vs. *IDH2*). No statistically significant difference was detected between the two groups (log-rank test, *P* = 0.973). The median time to blast clearance was 33 days for *IDH1*-mutated AML and 31 days for *IDH2*-mutated AML. The vertical axis represents the percentage of patients achieving blast clearance, and the horizontal axis represents days from induction start.

**Supplemental Figure 19. Effect of complete remission timing on Overall survival in IDH2-mutated AML.**
Kaplan–Meier analysis of overall survival among patients with IDH2-mutated AML, stratified by the timing of CRc achievement. *Early CRc* was defined as achieving CR or CRi at the end-of-induction bone marrow evaluation after the first induction cycle, whereas *late CRc* was defined as achieving CR or CRi after two or more induction cycles. Survival time was measured from the date of diagnosis to death, and patients were censored at the date of last follow-up if alive. The median overall survival (OS) was not reached in the early CRc group and was 16.6 months in the late CRc group (log-rank p = 0.40). Although not statistically significant, early responders demonstrated a trend toward improved survival.

**Supplemental Figure 20.** ***Overall survival in AML patients with IDH2 R140 vs. IDH2 R172 mutations.***Kaplan–Meier curves illustrate overall survival from the time of diagnosis, stratified by *IDH2* mutation subtype (R140 vs. R172). No statistically significant difference was observed between groups (log-rank test, *P* = 0.413). Median overall survival was 66.8 months for the *IDH2* R140 group; median survival was not reached for the *IDH2* R172 group. The vertical axis represents the probability of overall survival, and the horizontal axis represents months from diagnosis.

**Supplemental Figure 21.** ***Overall survival in AML patients with IDH1 vs. IDH2 mutations.***Kaplan–Meier curves illustrate overall survival from the time of diagnosis, stratified by *IDH* mutation type (*IDH1* vs. *IDH2*). No statistically significant difference was observed between the two groups (log-rank test, *P* = 0.703). Median overall survival was 44.4 months for the *IDH1*-mutated group; median survival was not reached for the *IDH2*-mutated group. The vertical axis represents the probability of overall survival, and the horizontal axis represents months from diagnosis.

**Supplemental Figure 22.** **Distribution of ELN 2022 risk classifications among patients with *IDH2*-mutated AML**.

Stacked bar plots depict the proportion of patients in each ELN 2022 risk category (Favorable = 0, Intermediate = 1, Adverse = 2) stratified by *IDH2* subtype (R140, n=23; R172, n=8). The distribution of risk categories was not significantly different between subtypes (Chi-square test, p = 0.77).
